# On the State of Lunatic Asylums in Ireland

**Published:** 1848-01-01

**Authors:** 


### STATE OF THE LUNATIC ASYLUMS IN IRELAND.

It was our intention to have entered fully into the consideration of this subject, with the view of forcing upon public and legislative attention the melancholy condition of some of the principal institutions for the reception of the insane in the sister kingdom. As we ponder over the details before us we can hardly conceive such a state of things to exist in a civilized state in the nineteenth century. In this country, where the legislature in its wise enactments enforces the residence of a qualified medical practitioner in every establishment where the number of inmates amounts to 100, will our readers believe—can they credit the fact? —that in Ireland there are public asylums for the insane, containing upwards of FOUR HUNDRED LUNATICS, without there being one resident medical officer in the establishment! Is this not disgraceful to a Christian country and a Christian government? We maintain that no philosophic or successful system of treatment, medical or moral, can be carried into operation in any establishment, in which acute cases of insanity are admitted, where a medical man does not reside. It is the duty of parliament to enforce the residence of a qualified medical man in every asylum.

Unless the medical officer-in-chief resides in the hospital; unless he be empowered to meet all difficulties as they arise by the possession of clear, definite, and unquestioned, but strictly responsible authority, a lunatic asylum under the best non-medical management is not an hospital for the treatment and cure of the insane, but with its sanded floors, clean beds, and whitewashed walls, is merely a well-regulated jail, holding within its walls patients or prisoners, who must be guarded as best they can by resident officers, hampered with restriction, and cramped in their authority; circumstances which reduce *them* to cyphers, and the *patients* or *prisoners* (it is unnecessary to cavil with either term) ultimately to incurable insanity!

In a great surgical or medical hospital for the treatment of bodily disease, which is plain, palpable, and often visible, should a patient be taken suddenly ill, so as to require immediate relief, a medical officer is at once sent for or at hand; but in our Irish Lunatic Asylums, devoted or supposed to be devoted to the treatment and cure of insanity, who ever dreams of sending for its solitary medical attendant to visit a patient suddenly excited, or bursting out into a furious paroxysm?—a matter of hourly occurrence in a great hospital. Yet these attacks are clear, plain, and unequivocal symptoms of cerebral disease or excitement, involving in their results, and repeated but neglected attacks, the loss of that which is of equal value to life—reason! Let it be taken for granted, that even in such a case the visiting physician should be sent for to the distance of a mile, the usual space between the asylums and the medical officer; he may be ten miles off at a private call, or by a special rule introduced for his personal convenience, be absent on his own leave for a fortnight altogether, so that patients are then thrown on the care of such casual medical friends as may give a call three times a week. Thus restraints are unavoidable, thus seclusion is necessarily resorted to, the results of which are frequently worse than those which follow restraint.

> "Quo nunquam radiis oriens medius ve cadens ve

> Phœbus adire potest nebulæ caligine mistæ

> Exhalantur humo, dubiœque crepuscula lucis

> Quo cubat ipse miser, Membris languore solutis

> Nec flecti cervix nec brachia reddere gestus

> Nec pes ire potest."

Had immediate and adequate authority been at hand; had a resident medical director, with free and unrestrained powers, existed, as he ought to exist, in the asylum, mild medical, combined with other and equally efficacious measures, would to a certainty allay sudden cerebral excitement, prevent its recurrence, and probably hasten a cure. Instead of that, an officer with no defined powers, no medical authority or knowledge, with a consciousness that he is doing what may be undone, and thus be exposed to the humiliation of official inferiority, is called on to interpose. Restraint alone, or seclusion, offers a ready subterfuge to escape from the difficulty, and as a consequence, one fit of excitement follows on the footsteps of another, until reason becomes, from the inability to bear up against the struggle, permanently unsettled; the usual consequences close the scene, and thus fill the Irish asylums with an overwhelming mass of despair, to remedy which, an additional number of *similarly conducted*

asylums are recommended, and will of course be called into existence in due time and place.

Another and an equally fatal result of the non-medical system is deserving notice.

By rules specially and studiously framed, so as to loosen all bonds of responsibility and order (*vide* rules 42 and 50), the visiting physician can attend at any hour it may suit his taste or convenience, or private interests; suppose (an ordinary matter) that he visits at three, four, five, or even later in the day. He prescribes for such as he may think fit, and departs. The apothecary must then be sent for, half a mile or a mile off; he has to compound and dispense his medicines, to apply blisters, to cup, bleed, &c., &c. The patients may be going to or actually in bed at the time these things have to be done. As lunatics are far different from any other class of patients, opposition, annoyance, or ill-humour, on the part of one patient at an unseasonable time, in a room where eighteen or twenty may be at rest, or in a cell or a corridor where fifteen or sixteen other patients may be settled for the night, excites the others. Waistcoats must be resorted to for the purpose of keeping on appliances which should have been attended to at ten or eleven o'clock, a.m. The consequences are torment or opposition; and a scene of disorder in the entire establishment during the rest of the night perfectly indescribable.

The mischievous consequences of *irregular* medical attendance do not end even here. The medical attendant, as before observed, under the provisions of rules specially made for his convenience, can attend at any hour that may suit his private advantage, or be accommodated to his other avocations; but subordinate servants of a great institution, engaged in attending on the sick, the wards, or walking rooms, must obtain leave of absence in *due time*, in *regular succession*, and at *stated hours*, otherwise no establishment could be conducted with order, or an approach to discipline. They are or may be absent when the medical attendant pays his desultory visit, and of course no satisfactory information can be given with anything like correctness, respecting the chief and essential duties relating to the inmates. How different would all this be? How little noise, confusion, or irregularity would exist, were the medical director of the asylum regularly to pay his professional visit at ten o'clock, a.m., accompanied three or four times a week by the visiting or consulting medical attendant, cheering some, comforting and advising all! How different from the present clumsy, noisy, bustling, and hurried mode of attending to the mental wants of hundreds!

When a visiting physician sees a patient, or a number of patients, in his hurried visits, who by a thousand devices know full well the hands where authority rests, he may order them a certain kind of treatment, according to his then view of their condition, and depart; they become, on his withdrawal, and with him the withdrawal of all supreme authority, restive, unmanageable, mischievous, or dangerous, in the extreme. They must then be controlled as best they can by those who, when the visiting physician again comes in a day or two, find their view corrected and their authority superseded, as it is most likely, nay almost certain, that the patients bow in cunning to his superior powers, make all kinds of professions of amendment, and gain their point. He departs as usual. The same game is played over again; hostility, moreover, is engendered in the minds of patients towards those who, to protect themselves, must resort to restraint or seclusion, while, in the meantime, as far as the patients are concerned, the fable of Penelope's web is re-acted, with this melancholy difference, that the thread not of flax, but of reason, is frequently snapped asunder for ever in this game of divided and irresponsible authority.

We subjoin below a tabular statement of the various asylums in Ireland, illustrative of the preceding remarks. In our next number, we hope to return to this important subject.

*District Lunatic Asylums in Ireland.*

| | Population of District. | No. of Inmates. | Physicians or Surgeons attaché. | Physicians or Surgeons residing. |
|---|---|---|---|---|
| Armagh | 832,474 | 137 | 1 | None resides. |
| Belfast | 722,321 | 251 | 2 | One resides, one does not. |
| Londonderry | 831,578 | 214 | 1 | None resides. |
| Richmond | 810,984 | 289 | 3 | None resides. |
| Carlow | 605,169 | 193 | 1 | None resides. |
| Ballinasloe | 1,418,859 | 310 | 1 | None resides. |
| Limerick | 910,303 | 339 | 1 | None resides. |
| Maryboro' | 557,578 | 194 | 1 | None resides. |
| Clonmell | 411,578 | 137 | 1 | None resides. |
| Waterford | 196,187 | 127 | 1 | None resides. |
| Cork | 854,118 | 419 | 2 | None resides. |

*Private Asylums.*

| Name. | County. | No. of Patients. |
|---|---|---|
| Retreat | Armagh | 23 |
| Citadella | Cork | 17 |
| Lindville | ditto | 41 |
| Bellvue | Dublin | 33 |
| Bloomfield | ditto | 22 |
| Eagle Lodge | ditto | 17 |
| Tarnham | ditto | 46 |
| Finglass | ditto | 12 |
| Hampstead | ditto | 24 |
| Hartfield | ditto | 10 |
| Lisle | ditto | 6 |
| Bushy Park | Limerick | 1 |
| Anne Brook | Queen's County } | 13 |
| Woodville | ditto } | |
| Cheek Point | Waterford | 2 |

Very few of these have a resident medical officer.

*Local Lunatic Asylums.*

| | | |
|---|---|---|
| House of Industry | Dublin | 103 |
| Island Bridge | ditto | 196 |
| Lifford | Donegal | 7 |
| Kilkenny | Kilkenny | 50 |

